# Using the Health Literacy Questionnaire (HLQ) with Providers in the Early Intervention Setting: A Qualitative Validity Testing Study

**DOI:** 10.3390/ijerph17072603

**Published:** 2020-04-10

**Authors:** Catherine J. Leslie, Melanie Hawkins, Diane L. Smith

**Affiliations:** 1 MGH Institute of Health Professions, Department of Occupational Therapy, Charlestown, MA 02129, USA; DSmith2@mghihp.edu; 2 School of Health and Social Development, Faculty of Health, Deakin University, Burwood, Melbourne 3125, Australia; melanie.hawkins@deakin.edu.au

**Keywords:** validity, early intervention, Health Literacy Questionnaire (HLQ), health literacy

## Abstract

More than one in four parents in the United States of America (USA) have low health literacy, which is associated with reduced health equity and negatively impacts child health outcomes. Early intervention (EI) programs are optimally placed to build the health literacy capacity of caregivers, which could improve health equity. The health literacy of interdisciplinary EI providers has not previously been measured. This study used the Health Literacy Questionnaire (HLQ) with EI providers (*n* = 10) to investigate evidence based on response (cognitive) processes. Narratives from cognitive interviews gave reasons for HLQ score choices, and concordance and discordance between HLQ item intent descriptions and narrative data were assessed using thematic analysis. Results found scales with highest concordance for Scales 3, 6, and 9 (each 96%, *n* = 24). Concordance was lowest on Scale 5 (88%, *n* = 22), although still strong with only 12% discordance. Three themes reflecting discordance were identified: (1) Differences between Australian and USA culture/health systems; (2) Healthcare provider perspective; and (3) Participants with no health problems to manage. Results show strong concordance between EI providers’ narrative responses and item intents. Study results contribute validity evidence for the use of HLQ data to inform interventions that build health literacy capacity of EI providers to then empower and build the health literacy of EI parents.

## 1. Introduction

### 1.1. Early Intervention and Health Literacy

In the United States of America (USA), the Individuals with Disabilities Education Act (IDEA) Part C Early Intervention (EI) Program serves families of infants and toddlers from birth to age three with or at risk for developmental delay [1]. Low parent health literacy is a national problem in the USA [2,3], and is associated with both negative child health outcomes [4,5,6] and health equity [7]. Health literacy has been defined by the World Health Organization (WHO) as: “the cognitive and social skills which determine the motivation and ability of individuals to gain access to, understand and use information in ways which promote and maintain good health” [8]. Adding to equity concerns, Americans’ low health literacy is significantly associated with race, socioeconomic status, and level of education [3,7]. By addressing low parent health literacy, EI providers can begin to address health inequities and disparities [7]. A healthcare provider’s understanding of health literacy can help mitigate the negative impact of low health literacy, and has resulted in improved pediatric and adult health outcomes and safety [9,10,11], although health literate practice by American healthcare providers is far from universal [12,13,14,15].

Kelly and Haidet [16] reported that a “Lack of knowledge among providers regarding issues related to health literacy can substantially alter patient–provider communication and hinder benefits expected from medical care”. This finding is especially significant and relevant in the EI setting, which is based on a model of indirect services where EI providers work with parents to set meaningful functional goals and strategies for the parents to implement during their daily routines [17,18]. This model requires effective communication between EI providers and parents to realize the potential benefits of EI services.

If EI providers have low health literacy themselves, this could hinder their ability to support the families they work with. The Health Literacy Questionnaire (HLQ) scales reflect skills related to EI clinical practice as outlined in the DEC Recommended Practices by the Division for Early Childhood (DEC) of the Council for Exceptional Children, a national organization in the USA charged with making evidence-based practice recommendations for professionals that work with young children who have, or are at risk for, developmental delays or disabilities [18]. For example, one DEC recommended practice is: “Practitioners provide the family with up-to-date, comprehensive and unbiased information in a way that the family can understand and use to make informed choices and decisions” [18] (p. 10). Implementation of this recommended practice by EI providers could be supported by having health literacy skills themselves, such as the items included in the HLQ scales regarding ability to compare health information from different sources, determine if new health information is accurate, locate up to date information about a health problem, and read and understand health information.

This study is embedded in a broader EI provider study measuring the health literacy of EI providers to identify areas of low health literacy where intervention can support EI providers to better respond to the health literacy needs of families enrolled in the EI programs. Although interdisciplinary EI providers are ideally situated in the American healthcare system to help mitigate the negative effects of low parent health literacy on child outcomes, there is limited research about the health literacy of EI providers [19] and no health literacy tools have been tested with EI providers in this setting. The current study will inform the choice of the HLQ as an assessment tool to measure the construct of health literacy in the EI setting.

### 1.2. The Health Literacy Questionnaire

The HLQ has been in use since 2013 [20]. The HLQ consists of 44 items within nine scales, each of which measures a distinct construct of the multidimensional construct of health literacy as shown in Table 1. The development of the HLQ was guided by the aforementioned WHO definition of health literacy. The HLQ was designed for a range of purposes including describing population health literacy, and informing the development and measuring the outcomes of health literacy interventions. Included in the documents provided to a licensed user of the HLQ are item intent descriptions written by the HLQ developers for each item. The item intent document provides a description of the meaning or intent of each item. This allows comparison of item intent descriptions with data obtained from HLQ test-takers, such as their chosen scores and their narratives about why they chose those scores.

### 1.3. Qualitative Validity Testing and Cognitive Interviewing

Validity is defined in the Standards for Educational and Psychological Testing as “the degree to which evidence and theory support the interpretations of test scores for proposed uses of tests”, and “the process of validation involves accumulating relevant evidence to provide a sound scientific basis for the proposed score interpretations” [21]. Qualitative research with the HLQ has indicated that patients’ scores and interview narratives are in concordance with the HLQ item intent descriptions, which provide evidence the HLQ items and constructs are understood as intended [22]. However, validity refers to the extent to which an intended interpretation and use of scores is justified by empirical evidence and the theory of the construct being measured [21,23,24,25,26,27]. Therefore, validation is an iterative process that requires investigation into the validity of score interpretation and use in each new setting for each new purpose [21,23,24,25,26,27].

As the HLQ has not been used with EI providers in the EI setting to date, this study is a first step toward contributing evidence to an argument for the validity of HLQ data from interdisciplinary EI providers. The study design does not result in a definitive finding of whether the HLQ is now validated for use with this population, but rather is a first step in the accumulation of evidence to support interpretation of HLQ scores of EI providers. In response to the recent movement towards theory-based validity testing of patient-reported outcome measures, and an identified need for validity evidence from qualitative research methods [23,27,28], this study used cognitive interviews to investigate evidence based on the response processes [29,30,31] of EI providers working with families of children with a developmental delay in EI programs. The focus of this study is on the response processes of EI providers gathered through cognitive interviewing as they completed HLQ items. Response processes are the “cognitive processes engaged in by test takers”, which when analyzed, “can provide evidence concerning the fit between the construct and the detailed nature of the performance or response actually engaged in by test takers” [21]. Evidence based on response processes is “the interpretation of test items by respondents as measured against the intended interpretation or construct definition” [23], which in this study was the comparison of participants’ narrative data to HLQ item intent descriptions. Participants’ narrative responses give insight into their cognitive processes when understanding the item, recalling information needed to respond to the item, formulating their response, and then choosing the response option that most closely matches their sense of how the item relates to them and their situation [21].

Cognitive interviewing has been described as “an important qualitative tool for the testing, development, and evaluation of survey questionnaires” used for “applying qualitative research methods to the understanding of the functioning of survey questions” [32]. The underlying theory is that individuals are interviewed using think-aloud and/or probing techniques to gather in-depth information in the form of narrative data that sheds light on the cognitive processes of the interviewees. The primary goal of this study was to determine if EI providers are engaging with the HLQ items as intended to provide evidence for the validity of the interpretation of EI provider HLQ scores in the EI setting. Additionally, the study findings add to previous research supporting the use of the HLQ in clinical and public health settings.

This study addressed the following aim and research questions (RQ):

Specific Aim: To qualitatively examine the use of the HLQ to measure the health literacy of EI healthcare providers in the EI setting. Specifically, the study sought to generate evidence based on response processes to determine if EI providers in the USA engage with the HLQ items as intended by the HLQ developers.

Research Question 1: Is there a match between the HLQ item score chosen and the interview narrative data from EI providers?

Research Question 2: Is there concordance between the HLQ item intents and the interview narrative data from EI providers?

Research Question 3: What are the reasons for not matching or discordance between the interview narrative data from EI providers and the HLQ item intents and HLQ item scores chosen?

## 2. Materials and Methods

### 2.1. Study Design

The study used a qualitative research design using EI providers’ HLQ item scores and cognitive interviewing with concurrent probing as they completed scales of the HLQ. With concurrent probing, the interviewer uses verbal probing with the participant after each survey item rather than after they have completed the whole survey [32]. The study methods followed recommendations by Willis for cognitive interviewing and probing processes [33]. The HLQ was self-administered using a pen and paper. The study design allowed for:

- Comparison of EI providers’ chosen HLQ score on each item and their narrative responses (RQ1): coded as match, no match, or unclear.
- Comparison of EI providers’ narrative data to the HLQ item intent descriptions provided by the developers of the HLQ (RQ2): coded as concordant, discordant, or unclear.
- Qualitative thematic analysis of narrative data across participants to identify reasons for coding of no match or discordance between the HLQ item intents, the HLQ item scores chosen, and interview narrative data (RQ3).

### 2.2. Ethics

This project was approved by the Partners Human Research Committee, which is the Institutional Review Board of Partners HealthCare: Protocol #2019P001134. Before being interviewed, participants were reminded about the goals for the study and that all information would be kept confidential and in a secure location. Each participant received a $25 USA gift card to a major department store for participating.

### 2.3. Setting

The study took place at two EI centers in Massachusetts (MA), USA, which supports long-term plans for future application of the HLQ with EI providers. The centers reflect a convenience sample and were chosen based on connections through community partnerships and contacts at these centers by an author of the study (C.L.). EI centers provide home-based services to families of children with or at risk for a developmental delay, and employ healthcare providers from multiple disciplines, such as physical, occupational and speech-language pathologists, developmental specialists, and nurses [17].

### 2.4. Materials

#### Health Literacy Questionnaire (HLQ)

The HLQ was developed using a validity-driven approach [34] with initial testing in diverse samples of individuals in Australian communities [20,35]. The psychometric properties of the nine scales have shown robust construct validity and reliability [20]. Several studies estimated the reliability of the HLQ with ranges from 0.77 to 0.90 and 0.80 to 0.89, and confirmed the HLQ’s nine-factor structure [36,37]. The HLQ has also shown sound psychometric properties in other contexts [36,37,38,39,40,41,42]. Items in scales 1–5 are scored on a 4-point response option scale (strongly disagree, disagree, agree, strongly agree) and on a 5-point response option scale in scales 6–9 (cannot do or always difficult, usually difficult, sometimes difficult, usually easy, always easy).

### 2.5. Participants

EI providers were recruited by distribution of a flyer describing the study by the EI center directors to their staff. EI providers representing different healthcare disciplines were included. Eligibility criteria included being 18 years of age or older, currently employed by an EI center in MA, and having verbal, written, or online contact with families in the EI program. Priority was given to enrolling participants from more than one EI center and those representing demographic variety [43], especially regarding our demographic variable related to participants’ role at their EI center, which is relevant to the interdisciplinary EI setting and to long-term project plans to administer the HLQ to a variety of EI providers across multiple sites.

### 2.6. Data Collection

Participants gave verbal consent when the interview was scheduled and again at the start of the interview. In order to keep the length of each interview to a reasonable length of time for the participants, the nine scales of the HLQ were separated into two groups. Completing cognitive interviewing for all nine scales by each participant would be time consuming and a cognitive burden on participants. Participants were assigned alternately to either Group 1: HLQ scales 1, 2, 3, 6, and 7 or Group 2: HLQ scales 4, 5, 8, and 9, in the order they enrolled in the study. Demographic data was also collected. Semi-structured cognitive interviews were conducted by one interviewer (C.L.) and were completed in person with each participant using a pen and paper version of the HLQ. The procedure for the cognitive interviewing using concurrent probing included: Interviewer read an HLQ item to the participant, who also read the item on their paper HLQ and checked off their answer choice for that item; interviewer carefully observed participant for any signs of hesitation or difficulty answering; interviewer used verbal probes, such as “What thoughts came to mind when you were choosing your answer to this item?”, to engage with participant and elicited verbal responses about reasons for choosing that response option for their answer choice, understanding of key phrases in the items, and any difficulty answering the item; moved to next HLQ item and repeated until finished with items for scales for their grouping.

### 2.7. Data Analysis

There were five EI providers in each interview group for a total of 10 participants. Participants are identified in this study using a P and their study number i.e., P#01 to P#10. Thematic analysis was used for data analysis, and is defined as “a method for identifying, analyzing, and reporting patterns (themes) within data. It minimally organizes and describes the data set in rich detail” [44]. The six phases of thematic analysis outlined by Braun and Clarke [44] were used to analyze the data: (1) familiarizing yourself with your data; (2) generating initial codes; (3) searching for themes; (4) reviewing themes; (5) defining and naming themes; and (6) producing the report.

A deductive theoretical thematic analysis was used to identify patterns in the data in order to code for our specific research questions, rather than an inductive thematic analysis used to generate research questions [44]. The coding of the data involved three steps. Step 1: coding to determine if participants’ chosen HLQ score on each item matched their narrative response for that item; Step 2: coding to determine if participants’ narrative data were concordant with the HLQ item intent descriptions provided by the developers of the HLQ; and Step 3: thematic reduction to determine the reasons for participants’ interview narrative data not matching or not being concordant with the HLQ item scores chosen or the HLQ item intents.

In Step 1, for each item, the respondent’s chosen response option for that item, such as disagree or always easy, was compared to their narrative response for that item on the transcript. After assessing whether the narrative data supported the choice of score for that item, the researcher analyzing the data for this item then coded that item as match, no match, or unclear. For example, a code of match would be applied to an item with a score of usually difficult if the participant’s narrative described what had made that item difficult for them. Conversely, a code of no match was applied when the score chosen was not supported by the narrative data. A code of unclear was assigned if there was no narrative data associated with the item, or the narrative was extremely brief (e.g., “Yeah.”).

For Step 2, for each item, the item intent description was compared to the narrative data for that item in the transcript. The narrative was analyzed to determine if it reflected the key components of the item intent description. Each item was coded as concordant, discordant, or unclear. A code of concordant was assigned if the participant’s narrative clearly supported and was related to the ideas in the item intent description. A code of discordant was assigned when participant narrative reflected a different understanding of the item than the HLQ developers provided. Unclear was coded as described previously. For both Step 1 and Step 2, if the narrative provided by a participant for a particular item was shorter than needed to confirm a code of match or concordant the narrative for the other items included in that scale were examined and used in deciding on the coding for that item.

Finally, in Step 3, qualitative thematic analysis of narrative data across participants was completed to identify reasons for scores of no match or discordant between the HLQ item intents, the HLQ item scores chosen, and interview narrative data.

Data were collected for 10 participants with five participants providing a score and narrative data for each item across the nine scales of the HLQ based on participant assignment to either Group 1 (Scales 1, 2, 3, 6, and 7) or Group 2 (Scales 4, 5, 8, and 9). Each of the nine HLQ scales consists of four to six related items that make up the scale, and the HLQ has a total of 44 items. Resulting data that were coded included 219 pairs (1 missing item answer) of participants’ scores on an item and the corresponding narratives coded as match, no match, or unclear, and 220 pairs of participants’ narratives on an item and the corresponding item intents coded as concordant, discordant, or unclear. One researcher (C.L.) independently coded all 439 pairs, and the second researcher (M.H.) independently coded 20% of the pairs across all scales. Related to Step 1 and Step 2 above, consensus was achieved after consultation regarding any coding differences between the two researchers through discussion and comparison of notes taken on each item during coding. For the next step, thematic analysis of narrative data from Step 3 was completed to produce categories of reasons for items being not a match or discordant. Categories were then organized into themes with sub-themes.

## 3. Results

Results will be reported for each of the three research questions (RQs). Demographic data for participants are displayed in Table 2. The average age of participants (*n* = 10) was 29.5 years, all 10 were female, nine were White, four reported having a chronic health condition, all 10 had a university education, and participants represented eight different EI job roles.

**RQ 1.** Is there a match between the HLQ item score chosen and interview narrative data from EI providers? 

Of the 219 pairings of a participant’s choice of item score for each item and their narrative responses, 94% of pairs (*n* = 206) were coded as a match. See Table 3 for matching across the nine scales. Scale 1 (“Feeling understood and supported by healthcare providers”) had the lowest match rate (84% match) with two participants having narratives that did not support their scores on the same item about feeling supported by their healthcare provider. Participants had 100% matching across the 25 pairings for Scale 9 (“Understand health information well enough to know what to do”). A pair was coded as a match if the participant’s narrative supported the score they chose. For example, on item #9 pt2 (Scale 9) about following instructions from healthcare providers, P#02 chose the response option of usually easy and the narrative supported that score so it was coded as a match: “I would say ’usually easy’ because sometimes after I visit a healthcare provider, I don’t fully remember what I’ve been told. So, I’ll think that I that I know something, but if I didn’t like write it down or make sure that I was paying close attention, I could easily forget it. And then if they even wrote it down for me, I can’t read their handwriting. So, it’s sometimes, it’s usually easy, but not always. I have to be on my game to make it easy.”

**RQ2.** Is there concordance between the HLQ item intents and the interview narrative data from EI providers? 

There was concordance in 204 of the 220 pairings of item intents and narrative data (93%). See Table 3 for data across scales on numbers of concordant, discordant, and unclear coding of pairs of item intent descriptions with narrative data. Scale 3 (“Actively managing my health”), Scale 6 (“Ability to actively engage with healthcare providers”), and Scale 9 (“Understand health information well enough to know what to do”) had 96%. The lowest concordance, but still high, at 88% was for Scale 5 (“Appraisal of health information”).

A pair was coded as concordant if the participant’s narrative supported and reflected the item intent descriptions. For example, on item #6 (Scale 3) about spending time to manage health, P#07′s (agree) narrative reflected the item intent description and was coded as concordant: “Well, for me, what I’m thinking about is just more managing, wanting to continue to be healthy, and working out is, like, probably what comes to mind the most. I know that the question’s probably asking you for more than that, but that’s what it brings up for me and thinking about eating healthy and being active.”

**RQ3.** What are the reasons for not matching or discordance between the HLQ item intents, the HLQ item scores chosen, and the interview narrative data from EI providers?

Analysis of RQ3 data for evidence based on response (cognitive) processes identified three themes for the reasons for no match or discordance between the item intent descriptions, the item scores, and the interview narrative data. Selected examples of participants’ narrative data for each theme are offered in the results. See Table 4 for additional examples to support each theme.

Themes for not matching or discordance:

Theme 1. USA culture/health system
Sub-theme 1.1.: Word “entitled”.
Sub-theme 1.2.: Difference in USA vs. Australian insurance.
Theme 2. The healthcare provider perspective
Sub-theme 2.1.: Answering based on provider perspective.
Sub-theme 2.2.: Only thinking of doctors or healthcare providers for support (not family/friends).
Theme 3. Respondents with no health problems to manage

Theme 1. USA Culture/Health System

This theme included two sub-themes: (1) the word “entitled”; and (2) differences in USA and Australian health insurance systems. Both reflect the differences between respondents in this study who chose their answers to items based on their context of living in and using the USA healthcare and insurance systems, and Australian participants in the HLQ development study answering in the context of the Australian healthcare and insurance systems.

Sub-theme 1.1. Word “entitled”

Item #16 pt2 (Scale 7) asks participants how easy or difficult they feel it is to find out which healthcare services they are entitled to. The data indicated that participants were not comfortable with the word ‘entitled’. For example, P#07 responded “Entitled is such a strong word. I think, I just feel it’s a little bit of a loaded word in my opinion. You know, but I find out which health care services. I don’t know what else you would say. That ‘you’re eligible for’ or ’you’re entitled to’ just seems a little loaded.”

Sub-theme 1.2. Differences in USA and Australian insurance systems

Medicare is the tax-funded Australian universal health insurance scheme [45]. All Australian residents are entitled to a range of health services and hospital care under the scheme. Access to health and hospital care in the USA is determined by user-pay insurance plans through private insurance companies. Health insurance clients are entitled only to the health services covered by their insurance plan, and this can sometimes be complicated to work out. Narrative data for item #16 pt2 (Scale 7) about services people are entitled to indicated that participants were responding to the item on the basis of what they might be allowed to claim from their health insurance company rather than, as Australian residents do, saying that they just ask their general practitioner [22]. For example, P#03 (always easy) stated “Yes. I know who I should be seen by or what services my insurance provides.” P#05 (sometimes difficult) reported “I think this kind of goes back to like the healthcare system. It’s ‘sometimes difficult’ because it varies I think between your health insurance. And sometimes I feel like I read one thing, or it’s actually really, it can be challenging to go into my like specific health care and find like this is how many visits I can go to or like this specific specialist isn’t covered or this procedure.”

Theme 2. The Healthcare Provider Perspective

Theme 2 is about how being a healthcare provider influenced the way participants scored the items. The two sub-themes are: (1) answering based on provider perspective; and (2) only thinking of doctors/healthcare providers for support. 

Sub-theme 2.1. Answering based on provider perspective

The narrative data indicated that some participants responded to the items from the perspective of thinking about their patients’ care rather than considering their own personal healthcare needs. For Item #3 pt2 (Scale 8; “Find information about health problems”), P#02 (usually easy) explained that “There’s some rare conditions that I feel like I haven’t always been able to find or easily find information on, because of the, like, just the rarity of what we’re [EI providers] dealing with. So I haven’t, it hasn’t always been very easy and it takes me a little bit of time.”

Sub-theme 2.2. Only thinking of doctors/healthcare providers for support

Scale 4 is about the support that people receive from family and friends. However, the narrative data indicated that participants were sometimes thinking about support from health professionals. P#06 (agree; item#3) stated “So, I know how to get in touch with my doctor but I don’t necessarily have, like, a team of health professionals.”

Theme 3. Respondents with no health problems to manage

Theme 3 describes the narrative data from participants who did not have health problems or did not feel in need of medical care or advice and were unsure about how to respond to some items. P#09’s narrative to Item #17 (Scale 1) about having the healthcare providers one needs to make health decisions was “Agree, but I feel like that implies that there’s always something that I need to do and I’m not really sure that that’s always the case.“ P#01 (disagree) stated “I would say disagree, but I think also I don’t feel like I have health problems” when asked about having enough information to manage her health (Item #10, Scale 2).

## 4. Discussion

The aim of this study was to qualitatively examine the use of the HLQ to measure the health literacy of EI healthcare providers in the EI setting. Specifically, the study sought to generate evidence based on response processes to determine if EI providers in the USA engage with the HLQ items as intended by the HLQ developers. This study found that cognitive interview narrative data from EI providers for each item supported the choice of HLQ item scores and, overall, these data were concordant with the HLQ item intent descriptions. These findings indicate that EI providers understand the items as intended and are engaging with the intended meanings of items when thinking about and making answer choices. These data provide qualitative validity evidence based on response processes to support the use of the HLQ with EI providers. To the best of our knowledge, this study is the first to qualitatively examine the use of the HLQ to measure the health literacy of EI providers by generating evidence based on response processes to determine if EI providers in the USA engage with the HLQ items as intended by the HLQ developers.

This study is important because healthcare provider understanding of health literacy can lead to better outcomes for patients [9,10,11]. In order to translate this evidence into the EI setting, we need to be able to measure the health literacy strengths and limitations of EI providers using a tool that reflects the multidimensional construct of health literacy as the HLQ does. This study contributes much needed evidence of validity for using HLQ data from EI providers to inform future interventions. To maximize health outcomes for infants and toddlers receiving EI services, EI providers use of health literacy knowledge and practices is needed to mitigate the known negative impacts of low parent health literacy [4,5,6]. With one in four American parents struggling with low health literacy [2,3], EI providers must provide services that meet the needs of parents with low health literacy so their children can benefit from EI services.

The outcomes of this study provide important evidence for minor caveats to do with one HLQ item from Scale 7 and interpretation of data from this item in the USA context. This includes the way the word ‘entitled’ is perceived in the USA, and the associated effects of the way that the USA health insurance system may cause respondents to think about the healthcare services they have a right to access. In the USA, the word ‘entitled’ is defined as “having a right to certain benefits or privileges or having or showing a feeling of entitlement” [46]. The word ‘entitled’ is sometimes used in a derogatory way in USA culture, as in the sentence “Some celebrities have an arrogant sense of entitlement” [46]. The HLQ was developed and initially tested with target samples in Australia [20]. There are differences between the Australian tax-funded Medicare universal health insurance scheme and the private user-pay insurance plans of the majority in the USA. The associated effects of these differences may cause respondents in the USA to think differently about the healthcare services they have a right to access than respondents in Australia. Participants responding to items in Scale 7 commented about needing to check with their insurance provider about being entitled to certain healthcare services, which may be different from how Australian respondents may interpret these same items. These word meaning and health insurance factors may introduce an element of construct-irrelevant variance in how people respond to the item, which may show a systematic difference between USA and Australian data for this item, and may need closer investigation. However, in the item intent description, the HLQ developers have accounted for the potential difficulty of using the word ‘entitled’ in other cultures and languages and for the differences in societies without government-funded healthcare. Given this difference is only for one item in Scale 7, the effects for the validity of interpretation of the overall score for Scale 7 for population studies are expected to be minimal.

Another important outcome of the study relates directly to administration of the HLQ with health care providers. This is that, in general, healthcare providers may need to be reminded to think about HLQ items from the point of view of their own health rather than that of their patients. Participants’ narrative data reflect difficulty choosing a response option and narrative that indicates their personal interactions with the healthcare system rather than as a healthcare provider. The HLQ has to date been used mainly to assess the health literacy of patients [22,35,41,42,47] or healthcare professions students [48,49,50]. The 10 study participants were healthcare providers actively working in the EI setting providing services to families and children with disabilities. A previous study using the HLQ found differences between clinicians’ and patients’ responses with clinicians thinking more about their responses and focusing more on the exact words used in an item than the patients [22]. The impact seen in these results of participants being healthcare providers suggests a need for additional emphasis for participants to consider each item from the viewpoint of their own health and healthcare experiences rather than those of their patients.

Although the HLQ was designed for people with and without health conditions, this study found that some healthcare providers are unsure of how to answer some items if they do not have health problems to manage. Participant narrative data found some difficulty answering some HLQ items when they felt they did not currently have health problems that they were trying to manage. Initial validation work with the HLQ involved calibration and replication samples from groups with health problems, including rheumatology patients, home and community care patients, and emergency department attendees [20]. Their replication sample purposefully targeted recruitment of people who were younger (with the goal of including people less likely to have chronic health conditions), but based on their final demographics, they were older and had more health problems than this study’s participants. Interestingly, other HLQ studies involving healthcare profession students [48,49,50] did not conduct qualitative studies, therefore it is unclear if they would have also had difficulty answering the HLQ items in relation to possibly having fewer health problems to manage.

A limitation of this study is that the findings from this sample of EI providers may not be generalizable to all other EI providers or other healthcare providers due to the makeup of the study sample, although the diverse range of clinical backgrounds of the participants is a positive factor. A further limitation of the study is that participants responded to the HLQ items by scales, based on the group they were assigned to, which resulted in answering the items in a different order than the format of the official questionnaire. This may have influenced how they answered each item. Each group was only given some of the scales, which would also have resulted in a less than typical arrangement of items. To keep the interviews to a manageable length, each item was answered by half the participants with a minimum of five answers per item.

A strength of the study is the use of the theoretically strong validity testing framework of the Standards to guide the purpose of the study and inform the interpretation of study results [21,23,27,28]. The Standards outline five sources of validity evidence needed when considering use of an assessment tool in a new setting with a new population: Evidence based on (1) test content; (2) response processes; (3) internal structure; (4) the relationship of the scores to other variables; and (5) validity and the consequences of testing. Test content of the HLQ has been investigated previously, primarily during the validity-driven development of the HLQ [20]. Evidence based on internal structure has been ongoing for the English HLQ and HLQ translations [37,38,39]. The HLQ consists of nine distinct, although related, scales in line with the definition of the Standards, HLQ studies for discriminant validity are placed within evidence for relations to other variables [20,36,38,39,40,41]. Evidence for the consequences of testing is a long-term effort requiring pre–post data. This is an ongoing process through the use of the HLQ in the Optimising Health Literacy and Access (Ophelia) process [51,52,53,54], which is now being widely implemented through independent projects and the WHO National Health Literacy Demonstration Projects (NHLDPs) [55]. This study with EI providers contributes evidence based on response processes for the interpretation of HLQ data from healthcare providers.

Population studies in the EI setting, including the larger over-arching study with EI providers in which this study is embedded, will contribute evidence of validity based on the internal structure of the HLQ and relations to other variables (i.e., discriminant validity). Use of the Standards’ framework helps organize and define the types of validity evidence that already exists and that needs to be generated for the use of the HLQ in new settings. By clearly presenting the validity evidence, other researchers can decide whether or not current evidence supports the use of the HLQ in their setting, or if further evidence needs to be generated by testing the HLQ in the new setting.

## 5. Conclusions

Results from this study show strong concordance between EI providers’ narrative responses and the HLQ item intents. Study findings contribute evidence to an argument for the validity of HLQ data for potentially informing interventions to build the health literacy capacity of interdisciplinary EI providers. Increased capacity of EI providers to be responsive to families’ health literacy needs will facilitate their work to empower and build the health literacy capacity of parents of children with special needs in EI. The outcomes of this study could potentially improve health equity for EI families and their children.

## Figures and Tables

**Table 1:** Health Literacy Questionnaire (HLQ) Scales.

The Nine Scales of the Health Literacy Questionnaire
Feeling understood and supported by healthcare providers (4 items). Having sufficient information to manage my health (4 items). Actively managing my health (5 items). Social support for health (5 items). Appraisal of health information (5 items). Ability to actively engage with healthcare providers (5 items). Navigating the healthcare system (6 items). Ability to find good health information (5 items). Understand health information well enough to know what to do (5 items).

**Table 2:** Profiles of early intervention (EI) providers (*n* = 10, all female).

Participant ID	EI Job Title (Years of EI Experience)	Education Level	Age in Years	Race	Chronic Health Condition(s)?	Self-Reported Health
P#01	DS (2)	Master’s	25	White	Yes	Excellent
P#02	OT (1)	Doctoral	24	White	No	Excellent
P#03	TL (3)	Master’s	27	Hispanic	Yes	Very good
P#04	OT (1)	Master’s	26	White	No	Good
P#05	MT (2)	Master’s	28	White	Yes	Good
P#06	DS (4)	Bachelor’s	29	White	No	Very good
P#07	Manager (11)	Bachelor’s	42	White	No	Very good
P#08	SLP (6)	Master’s	31	White	No	Good
P#09	RN (4)	Bachelor’s	28	White	No	Good
P#10	PT (4)	Doctoral	35	White	Yes	Good

DS: Developmental specialist; OT: Occupational therapist; TL: Team leader; MT: Music therapist; SLP: Speech language pathologist; RN: Registered nurse; PT: Physical Therapist. Health was self-reported as excellent, very good, good, fair, or poor.

**Table 3:** Matching and concordance rates across Health Literacy Questionnaire scales.

		Matching: Narrative Data and Item Score			Concordance: Narrative Data and HLQ Item Intent	
HLQ Scale	M N (%)	NM N (%)	U N (%)	C N (%)	D N (%)	U N (%)
Scale 1	16 (84%)	2 (11%)	1 (5%)	19 (95%)	-	1 (5%)
Scale 2	19 (95%)	1 (5%)	-	18 (90%)	1 (5%)	1 (5%)
Scale 3	24 (96%)	1 (4%)	-	24 (96%)	1 (4%)	-
Scale 4	24 (96%)	1 (4%)	-	23 (92%)	1 (4%)	1 (4%)
Scale 5	24 (96%)	1 (4%)	-	22 (88%)	3 (12%)	-
Scale 6	23 (92%)	1 (4%)	1 (4%)	24 (96%)	-	1 (4%)
Scale 7	28 (93%)	1 (3%)	1 (3%)	27 (90%)	1 (3%)	2 (7%)
Scale 8	23 (92%)	2 (8%)	-	23 (92%)	2 (8%)	-
Scale 9	25 (100%)	-	-	24 (96%)	1 (4%)	-
**HLQ** **Totals**	**206 (94%)**	**10 (5%)**	**3 (1%)**	**204 (93%)**	**10 (5%)**	**6 (3%)**

Percentages were rounded to nearest whole number. Due to rounding, totals may not equal 100%.M: match; NM: no match; U: unclear; C: concordant; D: discordant;

**Table 4:** Additional examples of not matching or discordance for themes 1, 2, and 3.

Theme	EI Provider Quotes
Theme 1. USA culture/health system	
Sub-theme 1.1. Word “entitled”	P#09 (Usually difficult) “I feel like that’s a usually difficult one, because I feel like it’s never really clear to anyone what you’re entitled to. So it’s kind of knowing. So unless you are like working in a hospital and know what people are entitled to because you have to [for your job as a healthcare provider] because you have to empower them and like that’s part of your job is to do that. But it’s hard to tell what you’re actually truly entitled to.”
Sub-theme 1.2. Difference in USA and Australian insurance systems	P#01 “Sometimes difficult only because of insurance. I was just trying to figure out what’s covered by insurance, and insurance is just confusing to me.” P#07 “I mean, it’s sometimes, depending on what insurance I’ve had at what point in time, sometimes it’s meant a referral from the primary care provider or you know them connecting me to a different department in order to have the care, to see they’re the right kind of provider that you need and that has always gone smoothly.” P#09 I: “How would you start to figure out if you’re entitled to that? P: I mean I feel like I would first go to my doctor and be like ‘is this something that you would help me with or do I have to go somewhere else?’ And if that’s not working, then the insurance company would kind of be the next place to go, but I feel like that’s hard as well.”
Theme 2. The Healthcare Provider Perspective	
Sub-theme 2.1. Answering Based on Provider Perspective	P#04 “I don’t know if I’m answering this right, but like I feel like because, being a clinician, I know so many other clinicians [at work] that when I stumble on new information or I’m trying to learn something in terms of maybe like speech or like other stuff that I wouldn’t be an expert in, I typically ask other health care providers their own experience, own like understanding, and own information base”. P#07 “I mean I think of this in relationship to EI [early intervention job]. That’s such a different question than it is in relationship to my own health”. P#09 “I mean I definitely am much better at children and helping families (at work) find the right health care that they need for their children. But like as an adult, I feel like that’s not an area that I’m well versed in in this part of the state.” P#10 “I agree. Probably agree more in my professional life with that than my personal life. If I go to appointments with a family in early intervention, then I’ll often help them ask those questions that maybe they don’t know to ask. But oftentimes, like for my own self, that can be challenging.”
Sub-theme 2.2. Only thinking of doctors/healthcare providers for support	P#04 “I know what questions to ask and I feel like, as a health care provider, I understand a lot of like, I don’t know, like what I need to gather, what information to gather, to find the supports that I need.”
Theme 3. Respondents with no health problems to manage	P#04 “If I need help? For me, I would say probably not health, because I don’t have any health issues, but if I need help, I would probably say right now like financially.” P#07 “I think the only reason I’m saying ‘agree’ instead of ‘strongly agree’ is more just you know I can see I don’t really have any really pressing health issues, and maybe if there were, then that might be something you’d want more information on. But as far as what I have now, I feel like I can effectively manage.”

P: participant. I: interviewer.
